# [Retracted] The effect of foxp3-overexpressing Treg cells on non-small cell lung cancer cells

**DOI:** 10.3892/mmr.2025.13644

**Published:** 2025-08-04

**Authors:** Jiangzhou Peng, Zigang Yu, Lei Xue, Jiabin Wang, Jun Li, Degang Liu, Qiang Yang, Yihui Lin

Mol Med Rep 17: 5860–5868, 2018; DOI: 10.3892/mmr.2018.8606

Following the publication of the above paper, it was drawn to the Editors’ attention by a concerned reader that the western blot data shown in Fig. 2C on p. 5863, the cell adhesion assay data in Fig. 3C on p. 5864, and scratch-wound assay data shown in Figs. 3D and 4B on p. 5865 were strikingly similar to data appearing in different form in other articles written by different authors at different research institutes that had already been published elsewhere prior to the submission of this paper to *Molecular Medicine Reports*.

In view of the fact that the abovementioned data had already apparently been published previously, the Editor of *Molecular Medicine Reports* has decided that this paper should be retracted from the Journal. The authors were asked for an explanation to account for these concerns, but the Editorial Office did not receive a reply. The Editor apologizes to the readership for any inconvenience caused.

